# Relationships between autistic traits, taste preference, taste perception, and eating behaviour

**DOI:** 10.1002/erv.2931

**Published:** 2022-06-12

**Authors:** Na Chen, Katsumi Watanabe, Tatsu Kobayakawa, Makoto Wada

**Affiliations:** ^1^ Department of Rehabilitation for Brain Functions Research Institute of National Rehabilitation Center for Persons with Disabilities Tokorozawa Japan; ^2^ Faculty of Science and Engineering Waseda University Tokyo Japan; ^3^ Human Informatics and Interaction Research Institute National Institute of Advanced Industrial Science and Technology Tsukuba Japan

**Keywords:** autistic traits, eating behaviour, taste perception, taste preference

## Abstract

Individuals with autism spectrum disorder exhibit atypical taste perception and eating behaviours. However, little is known about the effect of autistic traits on eating behaviours in the general population. This study explored the relationships between autistic traits, taste preferences, taste perceptions, and eating behaviours among Japanese population using an online questionnaire survey. The results showed significant effect of autistic traits on eating behaviours, that people with higher autistic traits tended to have higher selective eating behaviours, such as increased sensitivity to food texture and mixed flavours. Moreover, selective eating behaviours were correlated with the preference for sour taste and aftertaste sensitivity. Those results suggest that eating behaviours can be influenced by the relationship between autistic traits, taste perceptions, and taste preferences. We discuss these results in the context of previous findings, and future investigations into the possibility of solving selective eating problems in individuals with autism.

## Key points

- Autistic traits were significantly correlated with eating behaviours.
- People with higher autistic traits tended to have higher selective eating behaviours, such as increased sensitivity to food texture and mixed flavours.
- People who like sour tastes tended to have less selective eating behaviours.
- People who are sensitive to aftertaste perception tended to have greater selective eating behaviours.
- Autistic traits, taste perceptions, and taste preferences play a role in the development of a number of selective eating behaviours.

## 1 INTRODUCTION

Autism spectrum disorder (ASD) is a neurobiological developmental disorder, characterised by impaired social communication, restricted and repetitive behaviours, interests or activities, and difficulties in sensory processing. Specifically, differences in sensory perception are often significantly correlated with symptom severity in social cognition (Baum et al., 2015; Foss‐Feig et al., 2012). Atypical sensory experiences, such as hyper‐ or hypo‐sensitivity to sensory input, are reported to occur in as many as 90% of individuals with autism and affect almost every sensory modality: vision, audition, smell, touch, and taste (Robertson & Baron‐Cohen, 2017). Among these, taste has been investigated far less than the other senses.

A growing number of studies have reported high rates of atypical eating behaviours in individuals with ASD (Petitpierre et al., 2021). Atypical eating behaviours, such as food selectivity, food refusal, limited food preference, and high‐frequency single food intake are common eating difficulties among individuals with ASD (Cermak et al., 2010; Kral et al., 2013; Kuschner et al., 2015; Marí‐Bauset et al., 2014; Vissoker et al., 2015). It is well known that eating is a complex behaviour, which involves both external sensory inputs and internal processing. The external multisensory experience of eating relies on various sensory modalities, including visual appearance of food, auditory perception during eating, gustatory, olfactory, and tactile perception with food textures. Individuals with ASD exhibit different sensory processing (hyper‐ or hypo‐sensitivity to external sensory inputs) and impairment in multisensory integration (including subjective experience of internal sensation processing), which may lead to atypical eating behaviours (DuBois et al., 2016; Feldman et al., 2018; Kinnaird et al., 2019, 2020; Petitpierre et al., 2021; Tavassoli et al., 2014; Westwood & Tchanturia, 2017). To date, little is known about the effect of autistic traits on eating behaviours in relation to sensory information processing of food, such as visual appearance, smell, taste, texture, mixed flavours, and reactivity in interoceptive awareness. Autism spectrum disorder is generally understood as the extreme end of the quantitative distribution of autistic traits in the general population (Baron‐Cohen et al., 2001; Constantino, 2011; Westwood et al., 2016). Thus, understanding the effect of autistic traits on eating behaviours in general population may provide a better perspective on food selectivity and eating problems in individuals with autism.

Studies have shown that individuals with ASD exhibit atypical responses to taste perception (Boudjarane et al., 2017). For example, individuals with ASD are less accurate than matched controls in identifying basic tastes (Bennetto et al., 2007; Tavassoli & Baron‐Cohen, 2012). In contrast, a few studies have reported that there is little difference in taste detection thresholds between individuals with ASD and controls (Bennetto et al., 2007; Damiano et al., 2014). Taste perceptions, such as aftertaste perception (i.e., after swallowing, a coat of food is left behind in the mouth leading to the perception of ‘aftertaste’), taste recognition, taste detection, and sensation to mixed flavours are critical for food preferences and eating behaviours (Chamoun et al., 2018; Kinnaird et al., 2018). However, little is known about the effect of autistic traits on taste perception.

Taste preference is an important determinant of food choices and eating behaviours. People generally prefer tastes that signal beneficial nutrients, such as umami and sweet tastes (signalling calories), and dislike bitter and sour tastes (signalling poison) (Drewnowski, 1997; Scott, 1992; Ventura & Worobey, 2013). However, little has been known on the effect of autistic traits on basic taste preferences. Only one study has examined taste preferences in autistic perception. Damiano et al. (2014) reported that individuals with ASD show a similar preference for sweet taste as matched controls, suggesting that hedonic responses to sweet taste are relatively intact in ASD. Greater research is needed to explore relationships between autistic traits and taste preferences. Furthermore, taste preferences can directly influence eating behaviours (e.g., higher preference for sweet taste is associated with higher consumption of sweet foods; Mennella et al., 2014). Thus, selective eating behaviours may result from a preference for specific tastes.

Based on the previous findings, the current study aimed to explore relationships between autistic traits, taste perception, taste preference, and eating behaviours in the general population using an online questionnaire survey. The autistic traits were measured by a Japanese version of the autism spectrum quotient (AQ‐10) questionnaire survey (Kurita et al., 2005; Maeda et al., 2017). The AQ‐10 is a short version of the self‐reported AQ‐50 questionnaire developed for a brief and sensitive screening for ASD (Allison et al., 2012; Booth et al., 2013; Westwood et al., 2016). We hypothesised that autistic traits could affect taste perceptions, taste preferences, and eating behaviours due to the atypical sensory experiences. Moreover, eating behaviours could be influenced by taste preferences and taste perceptions.

## 2 METHODS

### 2.1 Participants

Ninety‐six Japanese individuals (57 females; 67 participants were between the age 15–29 years, 21 participants were between 30 and 49 years, and eight participants were above the age of 50 years) participated in an online questionnaire survey. A prior power analysis determined that a sample of 82 individuals would be sufficient to detect a correlation coefficient of 0.3 with an alpha of 0.05, and a power of 80% (Bujang & Baharum, 2016). We collected more data in case of missing inputs and errors. The participants were mainly from research participant pools at the National Rehabilitation Centre for Persons with Disabilities and Waseda University. Three participants who filled out the questionnaire with missing responses and two with duplicate responses were excluded. Hence, 91 participants' responses were finally used for data analysis. Nine of the participants were diagnosed with developmental disorders (five with ASD, one with learning disorder (LD), one with intellectual disability), other psychiatric disorders (one with depression, one with panic disorder). One participant reported ‘not sure (undiagnosed/suspected)’ about developmental disorders. One ASD participant had additional diagnoses of attention deficit hyperactivity disorder and depression; the LD participant had an additional diagnosis of cyclic vomiting syndrome. All the participants were Japanese speakers and agreed to participate in the questionnaire survey. This study was reviewed and approved by the ethics committee of the National Rehabilitation Centre for Persons with Disabilities (2020‐082).

### 2.2 Materials and procedure

This study was conducted using Google Forms. Participants undertook four sequential sessions (taste preference, eating behaviour, taste perception, and the autism spectrum quotient (AQ‐10) survey) (Kurita et al., 2005; Maeda et al., 2017). At the beginning of the online questionnaire survey, participants provided their consent to participate, and their demographic information, including age range, sex, diagnosed developmental disorder, and birthplace.

In the taste preference session, participants were instructed to rate how much they liked each of the basic taste words (i.e., sweet, sour, salty, umami, and bitter) on a five‐point Likert scale, arranged horizontally from left to right: (1) not like it at all, (2) a little dislike, (3) neutral, (4) a little like, and (5) like it very much.

In the session on eating behaviour, participants were asked to indicate the degree to which the content of each item best fits them on a five‐point Likert scale, arranged horizontally from ‘strongly disagree’ to ‘strongly agree.’ It included 19 items on eating behaviours related to the sensory experience of food, for for example, visual appearance (two items), smell (one item), texture (three items), taste (two items), mixed flavours (two items); interoception (two items); and eating habits with food selectivity (seven items) (Table 1). These items were developed based on the previous studies and interviews with the parents of children with ASD (Tabe & Takahashi, 2015; Takahashi & Masubuchi, 2008).

**TABLE 1:** Spearman's correlation coefficients between eating behaviours and autism spectrum quotient (AQ‐10) scores (ρ)

	Items	Correlation with AQ score
Visual appearance	1. There are some foods that I feel unpleasant or scared just looking at it;	0.13
	2. There are some foods I can't eat because I don't like the shape or colour;	0.22
Smell	I can't eat strong smell food;	0.22
Food texture	1. When eating some food, the texture can be annoying and unpleasant;	0.38**
	2. There are certain textures that I don't like, like a squishy or rough texture;	0.30*
	3. I don't like mixed textures, like soft bread with crunchy cucumber;	0.11
Taste	1. There are certain tastes that I don't like, like sour and umami seasoning;	0.30*
	2. I don't like deeply seasoned food;	0.26*
Mixed flavours	1. I don't like to mixed tastes, so I tend to eat all the dishes before eating the main food rice (or vice versa);	0.44**
	2. I don't like mixed tastes, like mixture from sweet and sour;	0.33**
Interoception	1. I feel like I'm drinking water all the time;	0.32**
	2. I don't know what it feels like to be thirsty or hungry;	0.34**
Food selectivity	1. There are many foods that I dislike and limited foods I can eat;	0.25*
	2. I tend to eat the same food every day;	0.34**
	3. I can't eat hot food;	0.15
	4. Vegetables are not delicious;	0.08
	5. I can't eat stimulating food, such as carbonated drinks and spices;	0.16
	6. There are some foods that I do not eat.	0.16
	7. I can't eat food that tastes different from what I expected;	0.16

*Note*: Values displayed in bold and with *asterisks indicate the level of statistical significance* (**p < 0.01, *p < 0.05, after FDR correction for multiple testing).

Abbreviation: FDR, False discovery rate.

In the taste perception session, participants were asked to rate the degree to which the content of four items best fits them using a five‐point Likert scale, arranged horizontally from ‘strongly disagree’ to ‘strongly agree.’ Four items on taste perception (aftertaste, recognition, detection, and mixed flavours) were used (see Table 2).

**TABLE 2:** Spearman's correlation coefficients between taste perceptions and autism spectrum quotient (AQ‐10) scores (ρ)

	Taste perception	Correlation with AQ score
Aftertaste	After eating, the aftertaste can be annoying for a long time;	0.25*
Recognition	I Can recognise some taste even if it's very light;	0.16
Detection	I Can tell if there are some specific tastes in certain food;	0.12
Mixed flavours	I Feel the taste changes when different tastes are mixed together;	0.25*

*Note*: *Asterisks indicate the level of statistical significance* (*p < 0.05, after FDR correction for multiple testing).

Abbreviation: FDR, False discovery rate.

**FIGURE 1:**
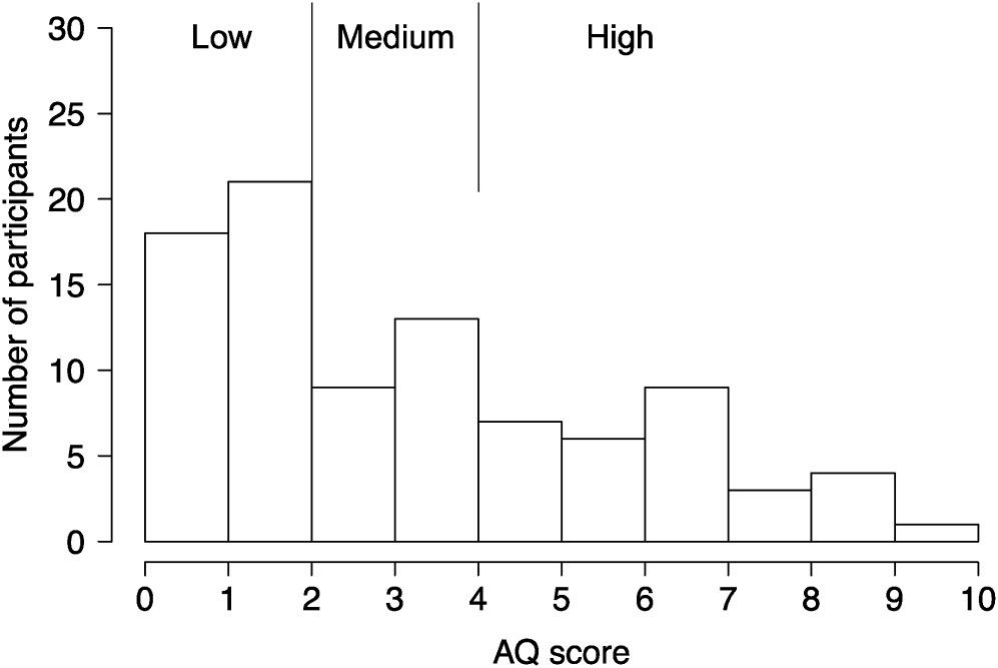
Distribution of participants' autism spectrum quotient (AQ‐10) scores

Finally, the participants completed the AQ‐10 questionnaire survey. Participants were asked to rate the degree to which the content of each item best fits them on a four‐point Likert scale (‘definitely agree,’ ‘slightly agree,’ ‘slightly disagree,’ and ‘definitely disagree’). For example, item 1 states, ‘I prefer to do things with others rather than on my own.’ Higher AQ scores indicated a greater magnitude of ASD traits. Distribution of AQ‐10 scores for the sample of 91 participants is shown in Figure 1. To further understand the effect of autistic traits, participants were divided into three groups as in a previous study (Chen et al., 2021). An AQ score of five was used as the criterion for the high AQ group (AQ ≥ 5; 30 participants), an AQ score of 2 as the criterion for the low AQ group (AQ ≤ 2; 39 participants), and participants with an AQ score between them were grouped into the medium AQ group (3 ≤ AQ ≤ 4; 22 participants).

### 2.3 Data analysis

Since the subjective ratings for taste preference, eating behaviour, taste perception, and AQ scores were not normally distributed (all *p*s < 0.05; Shapiro–Wilk's test), Spearman's rank correlation analysis was applied to examine the relationships between them. The false discovery rate (FDR) correction for multiple testing was used to adjust the *p‐values* and control for false‐positive results (Benjamini & Hochberg, 1995). A multivariate analysis of covariance (MANCOVA) with AQ group (low, medium, and high) as a fixed factor was conducted to reveal the effect of autistic traits on taste preference, taste perception, and eating behaviours. Further analysis adopted Principal Component Analysis (PCA) to explore the component structure underlying eating behaviours. Principal component analysis can be used to reduce high‐dimensional data sets to low‐dimensional sets of composite variables by grouping clusters of correlated variables. Groups of correlated variables are mapped onto one principal component to reduce the dimensionalities. After extracting the main PCA factors, Pearson's correlation analysis was used to examine correlations between the main factors of eating behaviours and AQ scores, taste preference, taste perception, separately. Data analyses were performed using R 4.0.0 software (R Core Team, 2020).

## 3 RESULTS

### 3.1 Taste preference and autistic traits

Spearman's correlation analysis showed no significant correlation between taste preferences and AQ scores (*p*s > 0.05, FDR corrected; Supplementary Table S1).MANCOVA showed no significant effect of AQ group on taste preferences, *F*(10, 170) = 1.15, Pillai's Trace = 0.13, *p* = 0.33, *η*_*p*_^2^ = 0.06. A repeated measurement Analysis of variance (ANOVA) revealed a significant main effect of taste preferences, *F*(4, 450) = 38.13, *p* < 0.001, *η*_*p*_^2^ = 0.25. Participants liked bitter taste less than the other four tastes (Tukey's Honestly significant difference (HSD), *p*s < 0.001), and liked sour less than sweet and umami tastes (Tukey's HSD, *p*s < 0.001).

### 3.2 Eating behaviour and autistic traits

Spearman's correlation analysis showed significant correlations between some eating behaviours and AQ scores (Table 1). Eating behaviours related to food texture, taste, mixed flavours, interoception, and food selectivity were positively correlated with AQ scores (*p*s < 0.05, FDR corrected). multivariate analysis of covariance analysis showed a significant main effect of AQ group on eating behaviours, *F*(38, 142) = 1.65, Pillai's Trace = 0.61, *p* = 0.02, *η*_*p*_^2^ = 0.31. Univariate ANCOVA showed that there were significant effects of AQ group on items related to mixed flavours, food textures, visual appearance, interoception, and food selectivity (*p*s < 0.05, FDR corrected; Supplementary Table S2). Thus, people in the high AQ group showed higher selective eating behaviours, such as less liking of mixed flavours, greater sensitivity to food texture, less interoceptively sensitivity, and greater persistent with specific food (Tukey's HSD, all *p*s < 0.05).

**FIGURE 2:**
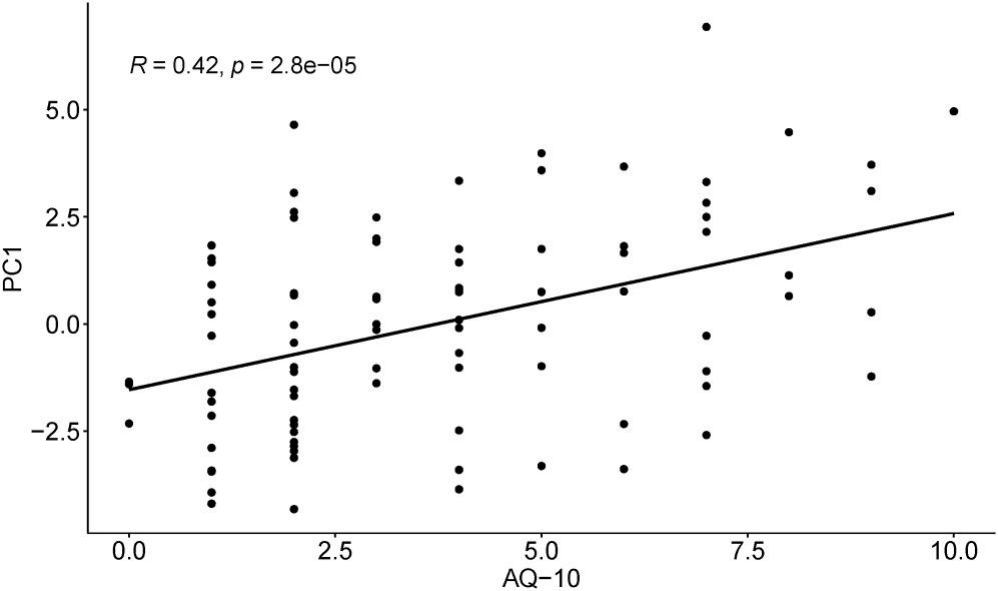
Correlation between the individual scores of PC1 and AQ scores

**FIGURE 3:**
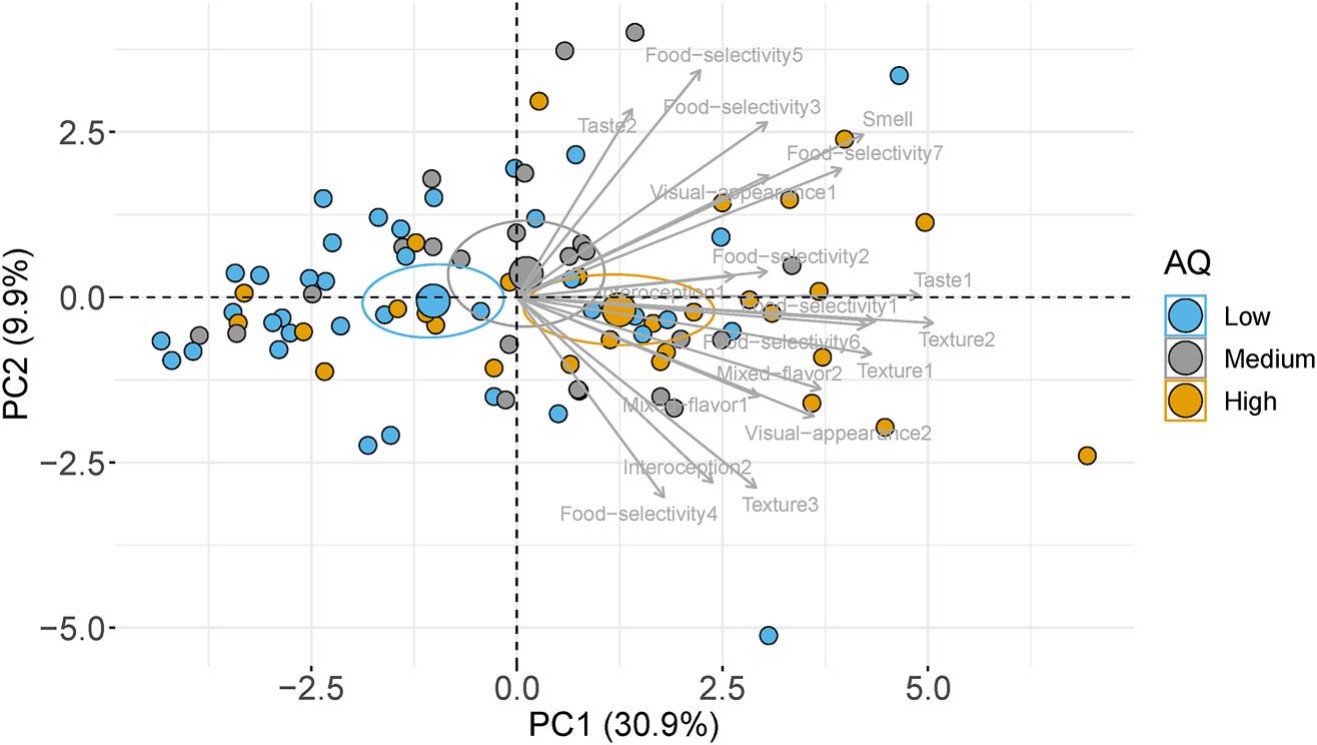
Principal component analysis (PCA) plot of variables (items in Table 1) and individuals in the three AQ groups. Note that confidence ellipses around the mean points of AQ groups. The low, medium, and high AQ groups are represented by the fill‐in colours blue, grey, and yellow, respectively

The ratings for the 19 items of eating behaviours were further subjected to PCA to extract the main factors underlying eating behaviours. Two factors were retained which explained 40.73% of the variance. The first factor (PC1; 30.86%) has strong variable loadings of food texture, food selectivity, smell, mixed flavours, and visual appearance (Supplementary Figure S1‐Contribution of variables to PC1). The second factor (PC2; 9.86%) has strong variable loadings of food selectivity, food texture, interoception, and smell (Supplementary Figure S1‐Contribution of variables to PC2). Pearson's correlation analysis showed a significant correlation between the individual scores of PC1 and AQ scores (*r* = 0.42, *p* < 0.0001; Figure 2), suggesting that participants with higher AQ scores tended to have higher selective eating behaviours. No significant correlation was observed between individual scores of PC2 and AQ scores (*r* = −0.01, *p* = 0.96). A one‐way ANOVA analysis also showed significant main effect of AQ group on PC1, *F*(2, 88) = 8.56, *p* < 0.01, *η*_*p*_^2^ = 0.16, indicating that participants in the high AQ group showed significant difference from participants in the low AQ group on the loading of PC1(Tukey's HSD, *p* < 0.001; see Figure 3).

### 3.3 Taste perception and autistic traits

Correlation analysis using Spearman's method showed certain significant correlations between taste perceptions and AQ scores (*p*s < 0.05, FDR corrected; Table 2). However, MANCOVA analysis showed no significant effect of AQ group on taste perceptions, *F*(8, 172) = 1.49, Pillai's Trace = 0.13, *p* = 0.16, *η*_*p*_^2^ = 0.06. Thus, the relationship between taste perceptions and AQ scores might be weak and could not be observed by covariance analysis with AQ groups.

### 3.4 Eating behaviours and taste preference

Correlation analysis showed negative correlations between preference for sour tastes and a few eating behaviours (*p*s < 0.05, FDR corrected; see Table 3). Participants who did not prefer a sour taste tended to be greater sensitive to food appearance, smell, food texture, taste, and mixed flavours, and had a higher food selectivity. No significant correlation was observed between preferences for other tastes and eating behaviours.

**TABLE 3:** Spearman's correlation coefficients between eating behaviours and taste preferences (ρ)

			Preference				
	Items		Sour	Salty	Sweet	Bitter	Umami
Visual appearance	There are some foods that I feel unpleasant or scared just looking at it;	−0.05		0.11	0.14	0.02	−0.02
	There are some foods I can't eat because I don't like the shape or colour;	−0.38**		−0.03	−0.04	−0.14	−0.18
Smell	I can't eat strong smell food;	−0.40**		−0.06	−0.09	−0.11	−0.09
Food texture	When eating some food, the texture can be annoying and unpleasant;	−0.16		0.16	0.16	−0.04	−0.07
	There are certain textures that I don't like, like a squishy or rough texture;	−0.36**		0.08	0.00	−0.07	−0.06
	I don't like mixed textures, like soft bread with crunchy cucumber;	−0.41**		0.01	−0.05	−0.07	−0.29
Taste	There are certain tastes that I don't like, like sour and umami seasoning;	−0.48**		0.14	−0.04	−0.06	−0.01
	I don't like deeply seasoned food;	0.11		−0.28	−0.13	0.04	−0.03
Mixed flavours	I don't like to mixed tastes, so I tend to eat all the dishes before eating the main food rice (or vice versa);	−0.13		0.02	−0.18	0.05	−0.03
	I don't like mixed tastes, like mixture from sweet and sour;	−0.37**		0.12	−0.19	−0.04	−0.22
Interoception	I Feel like I'm drinking water all the time;	−0.10		0.09	−0.01	0.07	−0.12
	I don't know what it feels like to be thirsty or hungry;	−0.05		0.03	0.07	−0.01	−0.03
Food selectivity	There are many foods that I dislike and limited foods I can eat;	−0.58**		0.06	−0.10	−0.11	−0.08
	I Tend to eat the same food every day;	0.05		0.08	0.01	0.18	−0.01
	I can't eat hot food;	−0.20		0.03	0.00	0.00	0.01
	Vegetables are not delicious;	−0.31*		−0.02	−0.03	−0.21	−0.05
	I can't eat stimulating food, such as carbonated drinks and spices;	−0.14		−0.05	0.07	−0.05	0.02
	There are some foods that I don't eat.	−0.46**		0.12	0.09	−0.15	−0.02
	I can't eat food that tastes different from what I expected;	−0.20		0.08	0.05	0.00	−0.03

*Note*: Values displayed in bold and with *asterisks indicate the level of statistical significance* (**p < 0.01, *p < 0.05, after FDR correction for multiple testing).

Abbreviation: FDR, False discovery rate.

**FIGURE 4:**
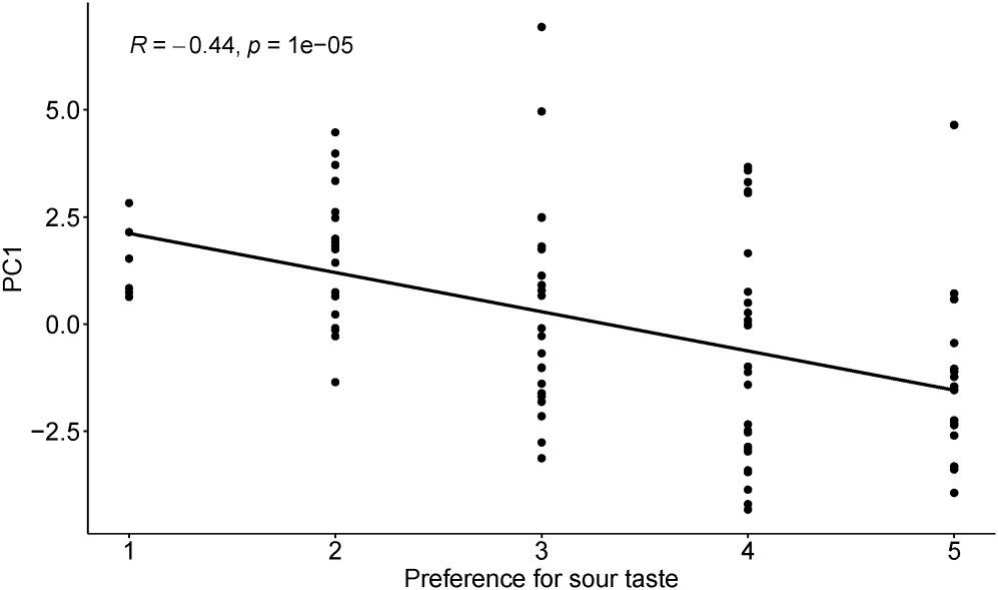
Correlation between the individual scores of PC1 and preference for sour taste

Further correlation analysis showed a significant correlation between the individual scores of PC1 and preference for sour taste (*r* = −0.44, *p* < 0.0001; Figure 4). No significant correlation was observed between the main factors and preferences for the other four tastes (*p*s > 0.05; Supplementary Table S3). One‐way ANOVA showed a significant main effect of sour preference (5 levels) on the individual scores of PC1, *F*(4, 86) = 5.83, *p* < 0.01, *η*_*p*_^2^ = 0.21. Participants in high preferences for sour taste (rating = 5, 4) showed significant differences in the loading of PC1 than participants in low sour preference (rating = 2; Tukey's HSD, *p*s < 0.01).

### 3.5 Eating behaviours and taste perception

Correlation analysis showed that there were significant correlations between a few eating behaviours and taste perceptions (*p*s < 0.05, FDR corrected; Table 4). The perception of aftertaste was significantly correlated with eating behaviours related to food texture, appearance, taste, and food selectivity. For example, people who were highly sensitive to aftertaste tended to be higher sensitive to food texture and taste and displayed greater selective eating behaviours. Moreover, people who felt sensitive towards taste perceptions tended to dislike deeply seasoned food (*ρ* = 0.41, *p* < 0.01, FDR corrected), and people who felt sensitive towards mixed flavours tended to dislike mixed flavours (*ρ* = 0.34, *p* < 0.01, FDR corrected).

**TABLE 4:** Spearman's correlation coefficients between taste perception and eating behaviours (ρ)

				Taste perception			
		Eating behaviours		Aftertaste	Recognition	Detection	Mixed flavours
Visual appearance		There are some foods that I feel unpleasant or scared just looking at it;	0.20		0.08	0.06	0.14
		There are some foods I can't eat because I don't like the shape or colour;	0.38**		0.13	0.13	0.16
Smell		I can't eat strong smell food;	0.23		0.12	−0.05	0.1
Food texture		When eating some food, the texture can be annoying and unpleasant;	0.54**		0.11	0.08	0.17
		There are certain textures that I don't like, like a squishy or rough texture;	0.40**		0.12	0.08	0.14
		I don't like mixed textures, like soft bread with crunchy cucumber;	0.28**		−0.06	−0.03	0.07
Taste		There are certain tastes that I don't like, like sour and umami seasoning;	0.52**		0.23	0.01	0.15
		I don't like deeply seasoned food;	0.15		0.41**	0.25	0.17
Mixed flavours		I don't like to mixed tastes, so I tend to eat all the dishes before eating the main food rice (or vice versa);	0.18		0.12	0.06	0.18
		I don't like mixed tastes, like mixture from sweet and sour;	0.23		0.01	−0.01	0.34**
Interoception		I Feel like I'm drinking water all the time;	0.15		0.03	0.11	0.14
		I don't know what it feels like to be thirsty or hungry;	0.21		0.16	0.13	0.11
Food selectivity		There are many foods that I dislike and limited foods I can eat;	0.41**		−0.03	0.04	0.14
		I Tend to eat the same food every day;	0.17		0.01	0.03	0.28
		I can't eat hot food;	0.25*		0.26	0.12	0.09
		Vegetables are not delicious;	0.25*		−0.05	−0.01	0.02
		I can't eat stimulating food, such as carbonated drinks and spices;	0.10		0.06	−0.03	0.11
		There are some foods that I don't eat.	0.41**		−0.10	−0.03	0.13
		I can't eat food that tastes different from what I expected;	0.14		0.04	0.02	0.09

*Note*: Values displayed in bold and with *asterisks indicate the level of statistical significance* (**p < 0.01, *p < 0.05, after FDR correction for multiple testing).

Abbreviation: FDR, False discovery rate.

**FIGURE 5:**
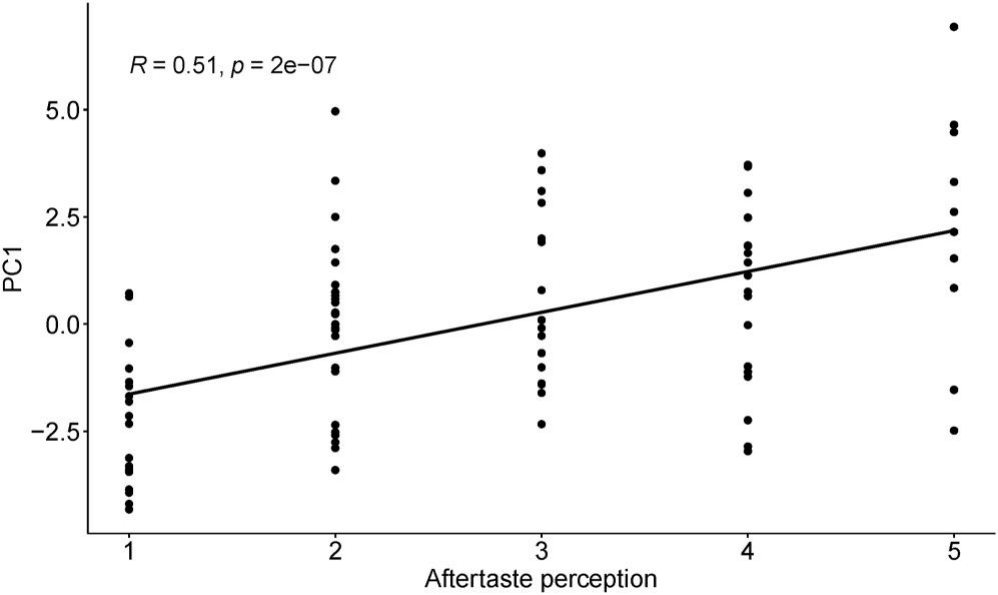
Correlation between the individual scores of PC1 and aftertaste perception

Further correlation analysis showed a significant correlation between individual scores of PC1 and aftertaste perception (*r* = 0.51, *p* < 0.0001; Figure 5), and mixed flavour perception (*r* = 0.25, *p* = 0.015). No other significant correlation was observed between the main factors and other taste perceptions (*p*s > 0.05; Supplementary Table S4). One‐way ANOVA on the effect of aftertaste (5 levels) on the individual scores of PC1 showed a significant main effect, *F*(4, 86) = 9.80, *p* < 0.01, *η*_*p*_^2^ = 0.31. Participants in higher sensitive to aftertaste (rating = 5, 4, 3, 2) showed significant differences from participants in the low sensitive to aftertaste in PC1 dimension (rating = 1; Tukey's HSD, *p*s < 0.01).

## 4 DISCUSSION

The present study investigated the relationships between autistic traits, taste preference, taste perception, and eating behaviour. The results showed that autistic traits were significantly correlated with eating behaviours. People with higher AQ scores tended to have higher selective eating behaviours, such as greater sensitivity to food texture, taste, mixed flavours; had less interoceptive awareness; and were greater selective about food. Furthermore, eating behaviours were significantly correlated with taste preferences and perceptions. Preference for sour taste was negatively correlated with a few selective eating behaviours, indicating that people who like sour taste tended to have less selective eating behaviours; while taste perceptions such as aftertaste perception, were positively correlated with a few selective eating behaviours, indicating that people who are sensitive to aftertaste perception tended to have greater selective eating behaviours. These results suggest that autistic traits, taste perceptions, and taste preferences may play a role in the development of a number of selective eating behaviours.

Studies have suggested that individuals with ASD have atypical eating behaviours, such as food selectivity, restricted food preferences, and repeated consumption of the same kind of food (Petitpierre et al., 2021). A few of these atypical eating behaviours are based on the food characteristics, such as food appearance, smell, texture, taste, and mixed flavours. In this study, it was observed that participants with higher AQ scores tended to be greater sensitive to food texture, taste, and mixed flavours, were less sensitive to interoception, and had greater selective eating behaviours. These results are in line with the previous findings, suggesting that hyper‐ and hypo‐sensitivity to sensory inputs and deficits in multisensory integration cause difficulties in sensory processing, leading to atypical eating behaviours in the ASD population (Cermak et al., 2010; Kinnaird et al., 2020; Kinnaird & Tchanturia, 2021; Kral et al., 2013; Kuschner et al., 2015; Ledford & Gast, 2006; Page et al., 2021; Vissoker et al., 2015). The core symptoms of ASD, restricted food preference (e.g., There are many foods that I dislike and limited foods I can eat) and repetitive eating behaviour (e.g., I tend to eat the same food every day) in participants with higher AQ scores are considered to be forms of restricted and repetitive behaviours, interests, or activities.

Specifically, the sensation of food texture is thought to be related to tactile perception in the mouth; hypersensitivity to tactile sensation is common in individuals with ASD (Puts et al., 2014, 2017; Tavassoli et al., 2016). Kuschner et al. (2015) also reported that individuals with ASD showed preferences for familiar foods and dislike for foods with particular textures and strong tastes. They suggested that broader sensory processing difficulties may partially explain a few of these food preferences. Besides, participants with higher AQ scores tended to have less interoceptive awareness, such as difficulty in identifying thirst or hunger, and excessive fluid drinking behaviours (e.g., ‘I feel like I'm drinking water all the time; I don't know what it feels like to be thirsty or hungry.’). These results were consistent with the previous findings, suggesting less sensitivity to interoception in individuals with ASD (Bremner & Regan, 1991; Fiene & Brownlow, 2015; Terai, Munesue, & Hiratani, 1999). Fiene and Brownlow (2015) reported that individuals with ASD have lower thirst awareness and are likely to consume large amounts of fluid. Terai et al. (1999) found that excessive drinking of water occurs highly often in children with autism than in mental retardation (controls). They suggested that this excessive water drinking behaviour might be related to a hypothalamic–pituitary dysfunction in autism, for that the thirst centre is reported to be located in the hypothalamus (Realmuto et al., 1990). One of the core symptoms in individuals with ASD is the atypical sensory processing and multisensory integration, in that hyper‐sensitivity and difficulties in binding sensory information may lead to increased sensitivity and lower preference or tolerance towards mixed flavours (Robertson & Baron‐Cohen, 2017). In addition, atypical sensory integration in individuals with higher autistic traits (Zhou et al., 2018) might be related to this tendency because mixed tastes inevitably are combinations of multiple basic tastes. We speculate that wider temporal binding windows of sensory stimuli in people with ASD would cause peculiar tastes due to mixed flavours in foods. Moreover, aftertaste perceptions were correlated with eating behaviours related to food texture and food selectivity. Participants who were sensitive to aftertastes tended to be highly sensitive to food texture and were highly selective regarding food. Perceptions of aftertaste and food texture are both related to the oral sensitivity and mouthfeel of the food. Hence, a higher sensitivity to aftertaste and food texture may lead to food selectivity. To solve selective eating behaviours, it might be helpful to improve the oral sensation experience of food, such as reducing aftertaste and increasing the pleasantness or acceptability of food textures. Future research may explore the effect of sensation on aftertaste and mixed flavours on related selective eating behaviours to address selective eating problems in both autistic and general populations.

Preference for the five basic tastes showed little correlation with autistic traits, indicating that autistic traits have little influence on affective responses to basic tastes. Damiano et al. (2014) also showed that individuals with ASD have an intact hedonic response to sweet tastes. Moreover, participants generally showed less preference for bitter tastes, consistent with the previous findings (Drewnowski, 1997; Reed & Knaapila, 2010; Sijtsema et al., 2012; Ventura & Worobey, 2013). We also observed that preference for sour taste was negatively correlated with eating behaviours. Participants who less preferred sour taste were greater selective in their eating behaviours. Previous studies have also shown that a higher preference or tolerance for sour taste indicates greater food acceptance and less selective eating behaviours (Frank & van der Klaauw, 1994; Liem et al., 2004). Liem et al. (2004) showed that children who prefer sour taste tend to experience greater dietary diversity, are willing to try unfamiliar foods, and prefer intense visual stimuli (e.g., bright colours). In contrast, Frank and van der Klaauw (1994) suggested that greater dietary diversity of foods may lead to a preference for higher levels of sourness intensity. Sour preference may be related to preference for stimulus, an overall thrill‐seeking behaviour that indicates a greater diversity of food acceptance and consumption. Moreover, sour preference has been associated with eating behaviours, such as consumption of fruits, particularly in children (Blossfeld et al., 2007; Liem et al., 2006; Liem & Mennella, 2003; Sijtsema et al., 2012). For instance, low intake of fruits and vegetables by preadolescent children may be related to their less preferences for bitter and sour tastes that are presented in fruits and vegetables (Sick et al., 2019). Based on these findings, it may be suggested that selective eating problems may be solved by increasing the preference or tolerance for sour taste. Future studies, to explore the mechanisms underlying the relationship between preference for sour taste and selective eating behaviours using psychophysical experimental methods are necessary.

One limitation of this study is that the eating behaviour questionnaire used has not been validated. The questions of eating behaviours were developed base on eating problems reported by parents of children with ASD and previous studies. Future studies may use validated eating behaviour questionnaires, such as the Adult Eating Behaviour Questionnaire (Hunot et al., 2016), to further reveal the effect of autistic traits on general eating behaviours. In addition, the self‐reported questionnaire was used to measure taste perception, which could not provide an objective measure. Future studies may use psychophysical methods with perceptual tastants to measure taste perceptions. Moreover, diagnoses in the sample were self‐reported and were not confirmed by a diagnostic review. Future studies should therefore aim to conduct perceptual experiments overcoming these limitations.

In summary, the present study complements the existing literature by revealing the relationships between autistic traits and taste preferences, taste perceptions, and eating behaviours. Autistic traits contribute towards development of eating behaviours such as preferences related to food textures, taste, mixed flavours, and interoceptive awareness. Furthermore, taste preferences and perceptions also contribute to a few selective eating behaviours such as preference for sour tastes and sensitivity to aftertastes. However, greater research is needed in this area to further examine taste perceptions, preferences, and eating behaviours in individuals with ASD and to reveal the mechanisms underlying autistic taste perceptions and related eating behaviours. The findings of this study can be suggested in solving eating problems faced by individuals with ASD and provide acceptable foods for diverse people.

## CONFLICT OF INTEREST

The authors declare that they have no known competing financial interests or personal relationships that could have appeared to influence the work reported in this paper.

**Abbreviations**
ASD: Autism Spectrum Disorder
AQ: Autism‐Spectrum Quotient
ANOVA: Analysis of variance
ANCOVA: Analysis of covariance
ADHD: Attention Deficit Hyperactivity Disorder
FDR: False discovery rate
HSD: Honestly significant difference
LD: Learning Disorder
MANCOVA: Multivariate analysis of covariance
PC: Principal component
PCA: Principal Component Analysis

## Supporting information

Supporting Information S1

## Data Availability

The data that support the findings of this study are openly available in OSF at https://osf.io/zgb52/.

## References

[erv2931-bib-0001] Allison, C. , Auyeung, B. , & Baron‐Cohen, S. (2012). Toward brief “red flags” for autism screening: The short autism spectrum quotient and the short quantitative checklist in 1, 000 cases and 3, 000 controls. Journal of the American Academy of Child & Adolescent Psychiatry, 51(2), 202–212. 10.1016/j.jaac.2011.11.003 22265366

[erv2931-bib-0002] Baron‐Cohen, S. , Wheelwright, S. , Skinner, R. , Martin, J. , & Clubley, E. (2001). The autism‐spectrum quotient (AQ): Evidence from Asperger syndrome/high‐functioning autism, males and females, scientists and mathematicians. Journal of Autism and Developmental Disorders, 31(1), 5–17. 10.1023/a:1005653411471 11439754

[erv2931-bib-0003] Baum, S. H. , Stevenson, R. A. , & Wallace, M. T. (2015). Behavioral, perceptual, and neural alterations in sensory and multisensory function in autism spectrum disorder. Progress in Neurobiology, 134, 140–160. 10.1016/j.pneurobio.2015.09.007 26455789 PMC4730891

[erv2931-bib-0004] Benjamini, Y. , & Hochberg, Y. (1995). Controlling the false discovery rate: A practical and powerful approach to multiple testing. Journal of the Royal Statistical Society: Series B, 57(1), 289–300. 10.1111/j.2517-6161.1995.tb02031.x

[erv2931-bib-0005] Bennetto, L. , Kuschner, E. S. , & Hyman, S. L. (2007). Olfaction and taste processing in autism. Biological Psychiatry, 62(9), 1015–1021. 10.1016/j.biopsych.2007.04.019 17572391 PMC2063511

[erv2931-bib-0006] Blossfeld, I. , Collins, A. , Boland, S. , Baixauli, R. , Kiely, M. , & Delahunty, C. (2007). Relationships between acceptance of sour taste and fruit intakes in 18‐month‐old infants. British Journal of Nutrition, 98(5), 1084–1091. 10.1017/s0007114507749231 17521470

[erv2931-bib-0007] Booth, T. , Murray, A. L. , McKenzie, K. , Kuenssberg, R. , O’Donnell, M. , & Burnett, H. (2013). Brief report: An evaluation of the AQ‐10 as a brief screening instrument for ASD in adults. Journal of Autism and Developmental Disorders, 43(12), 2997–3000. 10.1007/s10803-013-1844-5 23640304

[erv2931-bib-0008] Boudjarane, M. A. , Grandgeorge, M. , Marianowski, R. , Misery, L. , & Lemonnier, É. (2017). Perception of odors and tastes in autism spectrum disorders: A systematic review of assessments. Autism Research, 10(6), 1045–1057. 10.1002/aur.1760 28371114

[erv2931-bib-0009] Bremner, A. J. , & Regan, A. (1991). Intoxicated by water. The British Journal of Psychiatry, 158(2), 244–250. 10.1192/bjp.158.2.244 2012917

[erv2931-bib-0010] Bujang, M. A. , & Baharum, N. (2016). Sample size guideline for correlation analysis. World Journal of the Social Sciences Research, 3(1), 37–46. 10.22158/wjssr.v3n1p37

[erv2931-bib-0011] Cermak, S. A. , Curtin, C. , & Bandini, L. G. (2010). Food selectivity and sensory sensitivity in children with autism spectrum disorders. Journal of the American Dietetic Association, 110(2), 238–246. 10.1016/j.jada.2009.10.032 20102851 PMC3601920

[erv2931-bib-0012] Chamoun, E. , Mutch, D. M. , Allen‐Vercoe, E. , Buchholz, A. C. , Duncan, A. M. , Spriet, L. L. , Haines, J. , & Ma, D. W. L. , & Guelph Family Health Study . (2018). A review of the associations between single nucleotide polymorphisms in taste receptors, eating behaviors, and health. Critical Reviews in Food Science and Nutrition, 58(2), 194–207. 10.1080/10408398.2016.1152229 27247080

[erv2931-bib-0013] Chen, N. , Watanabe, K. , & Wada, M. (2021). People with high autistic traits show fewer consensual crossmodal correspondences between visual features and tastes. Frontiers in Psychology, 12, 3854. 10.3389/fpsyg.2021.714277 PMC8457010 34566793

[erv2931-bib-0014] Constantino, J. N. (2011). The quantitative nature of autistic social impairment. Pediatric Research, 69(8), 55–62. 10.1203/pdr.0b013e318212ec6e PMC3086844 21289537

[erv2931-bib-0015] Damiano, C. R. , Aloi, J. , Burrus, C. , Garbutt, J. C. , Kampov‐Polevoy, A. B. , & Dichter, G. S. (2014). Intact hedonic responses to sweet tastes in autism spectrum disorder. Research In Autism Spectrum Disorders, 8(3), 230–236. 10.1016/j.rasd.2013.12.003 24563662 PMC3927316

[erv2931-bib-0016] Drewnowski, A. (1997). Taste preferences and food intake. Annual Review of Nutrition, 17(1), 237–253. 10.1146/annurev.nutr.17.1.237 9240927

[erv2931-bib-0017] DuBois, D. , Ameis, S. H. , Lai, M. C. , Casanova, M. F. , & Desarkar, P. (2016). Interoception in autism spectrum disorder: A review. International Journal of Developmental Neuroscience, 52(1), 104–111. 10.1016/j.ijdevneu.2016.05.001 27269967

[erv2931-bib-0018] Feldman, J. I. , Dunham, K. , Cassidy, M. , Wallace, M. T. , Liu, Y. , & Woynaroski, T. G. (2018). Audiovisual multisensory integration in individuals with autism spectrum disorder: A systematic review and meta‐analysis. Neuroscience & Biobehavioral Reviews, 95, 220–234. 10.1016/j.neubiorev.2018.09.020 30287245 PMC6291229

[erv2931-bib-0019] Fiene, L. , & Brownlow, C. (2015). Investigating interoception and body awareness in adults with and without autism spectrum disorder. Autism Research, 8(6), 709–716. 10.1002/aur.1486 25808391

[erv2931-bib-0020] Foss‐Feig, J. H. , Heacock, J. L. , & Cascio, C. J. (2012). Tactile responsiveness patterns and their association with core features in autism spectrum disorders. Research In Autism Spectrum Disorders, 6(1), 337–344. 10.1016/j.rasd.2011.06.007 22059092 PMC3207504

[erv2931-bib-0021] Frank, R. A. , & van der Klaauw, N. J. (1994). The contribution of chemosensory factors to individual differences in reported food preferences. Appetite, 22(2), 101–123. 10.1006/appe.1994.1011 8037436

[erv2931-bib-0022] Hunot, C. , Fildes, A. , Croker, H. , Llewellyn, C. H. , Wardle, J. , & Beeken, R. J. (2016). Appetitive traits and relationships with BMI in adults: Development of the adult eating behaviour questionnaire. Appetite, 105, 356–363. 10.1016/j.appet.2016.05.024 27215837 PMC4990060

[erv2931-bib-0023] Kinnaird, E. , Norton, C. , Pimblett, C. , Stewart, C. , & Tchanturia, K. (2019). Eating as an autistic adult: An exploratory qualitative study. PLoS One, 14(8), e0221937. 10.1371/journal.pone.0221937 31465510 PMC6715205

[erv2931-bib-0024] Kinnaird, E. , Stewart, C. , & Tchanturia, K. (2018). Taste sensitivity in anorexia nervosa: A systematic review. International Journal of Eating Disorders, 51(8), 771–784. 10.1002/eat.22886 29984498 PMC6282513

[erv2931-bib-0025] Kinnaird, E. , Stewart, C. , & Tchanturia, K. (2020). The relationship of autistic traits to taste and olfactory processing in anorexia nervosa. Molecular Autism, 11(1), 1–10. 10.1186/s13229-020-00331-8 32276668 PMC7146886

[erv2931-bib-0026] Kinnaird, E. , & Tchanturia, K. (2021). Looking beneath the surface: Distinguishing between common features in autism and anorexia nervosa. Journal of Behavioral and Cognitive Therapy, 31(1), 3–13. 10.1016/j.jbct.2020.09.001

[erv2931-bib-0027] Kral, T. V. , Eriksen, W. T. , Souders, M. C. , & Pinto‐Martin, J. A. (2013). Eating behaviors, diet quality, and gastrointestinal symptoms in children with autism spectrum disorders: A brief review. Journal of Pediatric Nursing, 28(6), 548–556. 10.1016/j.pedn.2013.01.008 23531467

[erv2931-bib-0028] Kurita, H. , Koyama, T. , & Osada, H. (2005). Autism‐Spectrum Quotient‐Japanese version and its short forms for screening normally intelligent persons with pervasive developmental disorders. Psychiatry and Clinical Neurosciences, 59(4), 490–496. 10.1111/j.1440-1819.2005.01403.x 16048456

[erv2931-bib-0029] Kuschner, E. S. , Eisenberg, I. W. , Orionzi, B. , Simmons, W. K. , Kenworthy, L. , Martin, A. , & Wallace, G. L. (2015). A preliminary study of self‐reported food selectivity in adolescents and young adults with autism spectrum disorder. Research In Autism Spectrum Disorders, 15, 53–59. 10.1016/j.rasd.2015.04.005 26309446 PMC4545503

[erv2931-bib-0030] Ledford, J. R. , & Gast, D.L. , (2006). Feeding problems in children with autism spectrum disorders: A review. Focus On Autism And Other Developmental Disabilities, 21(3), 153–166.

[erv2931-bib-0031] Liem, D. G. , Bogers, R. P. , Dagnelie, P. C. , & deGraaf, C. (2006). Fruit consumption of boys (8–11 years) is related to preferences for sour taste. Appetite, 46(1), 93–96. 10.1016/j.appet.2005.11.002 16360976

[erv2931-bib-0032] Liem, D. G. , & Mennella, J. A. (2003). Heightened sour preferences during childhood. Chemical Senses, 28(2), 173–180. 10.1093/chemse/28.2.173 12588738 PMC2789429

[erv2931-bib-0033] Liem, D. G. , Westerbeek, A. , Wolterink, S. , Kok, F. J. , & De Graaf, C. (2004). Sour taste preferences of children relate to preference for novel and intense stimuli. Chemical Senses, 29(8), 713–720. 10.1093/chemse/bjh077 15466817

[erv2931-bib-0034] Maeda, Y. , Kaneyama, Y. , & Sato, H. (2017). Investigation on the autistic spectrum tendency of university student: Using AQ‐J‐10 (in Japanese). Kansai Psychological Research, 8, 23–29.

[erv2931-bib-0035] Marí‐Bauset, S. , Zazpe, I. , Mari‐Sanchis, A. , Llopis‐González, A. , & Morales‐Suárez‐Varela, M. (2014). Food selectivity in autism spectrum disorders: A systematic review. Journal of Child Neurology, 29(11), 1554–1561. 10.1177/0883073813498821 24097852

[erv2931-bib-0036] Mennella, J. A. , Finkbeiner, S. , Lipchock, S. V. , Hwang, L. D. , & Reed, D. R. (2014). Preferences for salty and sweet tastes are elevated and related to each other during childhood. PLoS One, 9(3), e92201. 10.1371/journal.pone.0092201 24637844 PMC3956914

[erv2931-bib-0037] Page, S. D. , Souders, M. C. , Kral, T. V. , Chao, A. M. , & Pinto‐Martin, J. (2021). Correlates of feeding difficulties among children with autism spectrum disorder: A systematic review. Journal of Autism and Developmental Disorders, 52, 1–20. 10.1007/s10803-021-04947-4 33666799

[erv2931-bib-0038] Petitpierre, G. , Luisier, A. C. , & Bensafi, M. (2021). Eating behavior in autism: Senses as a window towards food acceptance. Current Opinion in Food Science, 41, 210–216. 10.1016/j.cofs.2021.04.015

[erv2931-bib-0039] Puts, N. A. , Wodka, E. L. , Harris, A. D. , Crocetti, D. , Tommerdahl, M. , Mostofsky, S. H. , & Edden, R. A. (2017). Reduced GABA and altered somatosensory function in children with autism spectrum disorder. Autism Research, 10(4), 608–619. 10.1002/aur.1691 27611990 PMC5344784

[erv2931-bib-0040] Puts, N. A. , Wodka, E. L. , Tommerdahl, M. , Mostofsky, S. H. , & Edden, R. A. (2014). Impaired tactile processing in children with autism spectrum disorder. Journal of Neurophysiology, 111(9), 1803–1811. 10.1152/jn.00890.2013 24523518 PMC4044368

[erv2931-bib-0041] R Core Team (2020). R: A language and environment for statistical computing. R Foundation for Statistical Computing. Retrieved from https://www.R‐project.org/

[erv2931-bib-0042] Realmuto, G. M. , Jensen, J. B. , Reeve, E. , & Garfinkel, B. D. (1990). Growth hormone response to L‐dopa and clonidine in autistic children. Journal of Autism and Developmental Disorders, 20(4), 455–465. 10.1007/bf02216052 2279968

[erv2931-bib-0043] Reed, D. R. , & Knaapila, A. (2010). Genetics of taste and smell: Poisons and pleasures. Progress In Molecular Biology And Translational Science, 94, 213–240. 21036327 10.1016/B978-0-12-375003-7.00008-X PMC3342754

[erv2931-bib-0044] Robertson, C. E. , & Baron‐Cohen, S. (2017). Sensory perception in autism. Nature Reviews Neuroscience, 18(11), 671–684. 10.1038/nrn.2017.112 28951611

[erv2931-bib-0045] Scott, T. R. (1992). Taste, feeding, and pleasure. Progress in Psychobiology and Physiological Psychology, 15, 231–291.

[erv2931-bib-0046] Sick, T. , Rotter, J. M. , Reuter, S. , Kandambeth, S. , Bach, N. N. , Döblinger, M. , Merz, J. , Clark, T. , Marder, T. B. , Bein, T. , & Medina, D. D. (2019). Switching on and off interlayer correlations and porosity in 2D covalent organic frameworks. Journal of the American Chemical Society, 141(32), 12570–12581. 10.1021/jacs.9b02800 31251878

[erv2931-bib-0047] Sijtsema, S. J. , Reinders, M. J. , Hiller, S. R. , & Guàrdia, M. D. (2012). Fruit and snack consumption related to sweet, sour and salty taste preferences. British Food Journal, 114(7), 1032–1046. 10.1108/00070701211241608

[erv2931-bib-0048] Tabe, A. , & Takahashi, S. (2015). Developmental disabilities and other special needs in school meals: A survey of classroom guidance, special needs classes in Tokyo (in Japanese). Research Presentations at the Japan Education Association Conference, 74, 182–183.

[erv2931-bib-0049] Takahashi, S. , & Masubuchi, M. (2008). A study of real conditions and support of "hyper‐sensitivity and insensibility" of persons with asperger syndrome and high‐functioning autism: Needs survey of persons with asperger syndrome and high‐functioning autism (in Japanese). Bulletin of Tokyo Gakugei University Educational Sciences, 59, 287–310.

[erv2931-bib-0050] Tavassoli, T. , & Baron‐Cohen, S. (2012). Taste identification in adults with autism spectrum conditions. Journal of Autism and Developmental Disorders, 42(7), 1419–1424. 10.1007/s10803-011-1377-8 22006402

[erv2931-bib-0051] Tavassoli, T. , Bellesheim, K. , Tommerdahl, M. , Holden, J. M. , Kolevzon, A. , & Buxbaum, J. D. (2016). Altered tactile processing in children with autism spectrum disorder. Autism Research, 9(6), 616–620. 10.1002/aur.1563 26568449

[erv2931-bib-0052] Tavassoli, T. , Miller, L. J. , Schoen, S. A. , Nielsen, D. M. , & Baron‐Cohen, S. (2014). Sensory over‐responsivity in adults with autism spectrum conditions. Autism, 18(4), 428–432. 10.1177/1362361313477246 24085741

[erv2931-bib-0053] Terai, K. , Munesue, T. , & Hiratani, M. (1999). Excessive water drinking behavior in autism. Brain & Development, 21(2), 103–106. 10.1016/s0387-7604(98)00079-5 10206527

[erv2931-bib-0054] Ventura, A. K. , & Worobey, J. (2013). Early influences on the development of food preferences. Current Biology, 23(9), R401–R408. 10.1016/j.cub.2013.02.037 23660363

[erv2931-bib-0055] Vissoker, R. E. , Latzer, Y. , & Gal, E. (2015). Eating and feeding problems and gastrointestinal dysfunction in autism spectrum disorders. Research in Autism Spectrum Disorders, 12, 10–21. 10.1016/j.rasd.2014.12.010

[erv2931-bib-0056] Westwood, H. , Eisler, I. , Mandy, W. , Leppanen, J. , Treasure, J. , & Tchanturia, K. (2016). Using the autism‐spectrum quotient to measure autistic traits in anorexia nervosa: A systematic review and meta‐analysis. Journal of Autism and Developmental Disorders, 46(3), 964–977. 10.1007/s10803-015-2641-0 26542816 PMC4746216

[erv2931-bib-0057] Westwood, H. , & Tchanturia, K. (2017). Autism spectrum disorder in anorexia nervosa: An updated literature review. Current Psychiatry Reports, 19(7), 41. 10.1007/s11920-017-0791-9 28540593 PMC5443871

[erv2931-bib-0058] Zhou, H. Y. , Cai, X. L. , Weigl, M. , Bang, P. , Cheung, E. , & Chan, R. (2018). Multisensory temporal binding window in autism spectrum disorders and schizophrenia spectrum disorders: A systematic review and meta‐analysis. Neuroscience & Biobehavioral Reviews, 86, 66–76. 10.1016/j.neubiorev.2017.12.013 29317216

