# Supplementary material for: Relationships between autistic traits, taste preference, taste perception, and eating behaviour
Source: Eur Eat Disord Rev. 2022 Jun 12;30(5):628–40. doi: 10.1002/erv.2931 (PMC9545735; doi:10.1002/erv.2931)
Supplement: Supplementary file 1 — Supporting Information S1 [file ERV-30-628-s001.docx]

**Supplementary materials**

Table S1. Spearman’s correlation coefficients between the basic taste preference and AQ scores (ρ)

|  | | Sweet preference | Salty preference | Bitter preference | Sour preference | Umami preference |
| --- | --- | --- | --- | --- | --- | --- |
| AQ | ρ | -0.08  0.47 | 0.01  0.95 | 0.15  0.15 | -0.07  0.5 | -0.11  0.31 |
|  | *p* |  |  |  |  |  |

*Note:* *p* value before FDR correction for multiple testing.

Table S2. Results of univariate analysis of covariance on the effect of AQ group on eating behaviors

|  | Eating behaviors | F | *p* | η_p_^2^ |  |
| --- | --- | --- | --- | --- | --- |
| Visual appearance | | There are some foods that I feel unpleasant or scared just looking at it; | 0.37 | 0.69 | 0.01 |
|  |  | There are some foods I can’t eat because I don’t like the shape or color; | 5.85 | 0.004^**^ | 0.12 |
| Smell | | I can't eat strong smell food; | 2.31 | 0.10 | 0.05 |
| Food texture | | When eating some food, the texture can be annoying and unpleasant; | 6.34 | 0.003^**^ | 0.13 |
|  |  | There are certain textures that I don't like, like a squishy or rough texture; | 4.77 | 0.01^*^ | 0.10 |
|  |  | I don’t like mixed textures, like soft bread with crunchy cucumber; | 1.26 | 0.29 | 0.03 |
| Taste | | There are certain tastes that I don't like, like sour and umami seasoning; | 3.25 | 0.04 | 0.07 |
|  |  | I don’t like deeply seasoned food; | 3.36 | 0.04 | 0.07 |
| Mixed flavors | | I don't like to mixed tastes, so I tend to eat all the dishes before eating the main food rice (or vice versa); | 9.43 | 0.0002^**^ | 0.18 |
|  |  | I don’t like mixed tastes, like mixture from sweet and sour; | 6.39 | 0.003^**^ | 0.13 |
| Interoception | | I feel like I’m drinking water all the time; | 4.92 | 0.009^*^ | 0.10 |
|  |  | I don’t know what it feels like to be thirsty or hungry; | 3.18 | 0.05 | 0.07 |
| Food selectivity | | There are many foods that I dislike and limited foods I can eat; | 2.79 | 0.07 | 0.06 |
|  |  | I tend to eat the same food every day; | 5.05 | 0.008^*^ | 0.10 |
|  |  | I can't eat hot food; | 1.53 | 0.22 | 0.03 |
|  |  | Vegetables are not delicious; | 0.45 | 0.64 | 0.01 |
|  |  | I can't eat stimulating food, such as carbonated drinks and spices; | 0.55 | 0.58 | 0.01 |
|  |  | There are some foods that I don’t eat. | 1.37 | 0.26 | 0.03 |
|  |  | I can't eat food that tastes different from what I expected; | 1.63 | 0.20 | 0.04 |

*Note: Asterisks indicate the level of statistical significance* (^**^*p* < .01, ^*^*p* < .05, ﻿after FDR correction for multiple testing).

Table S3. Pearson’s correlation coefficients between the main PCA factors and taste preferences (r).

|  | Sweet preference | Salty preference | Bitter preference | Sour preference | Umami preference |
| --- | --- | --- | --- | --- | --- |
| PC1 | 0.09 | 0.06 | -0.07 | -0.44^**^ | -0.12 |
| PC2 | -0.07 | 0.03 | 0.13 | 0.12 | 0.05 |

*Note: Asterisks indicate the level of statistical significance* (^**^*p* < .01, ﻿after FDR correction for multiple testing).

Table S4. Pearson’s correlation coefficients between the main PCA factors and taste perceptions (r).

|  | Aftertaste | Recognition | Detection | Mixed flavors |
| --- | --- | --- | --- | --- |
| PC1 | 0.51^**^ | 0.16 | 0.07 | 0.25^*^ |
| PC2 | -0.14 | 0.17 | 0.00 | -0.05 |

*Note: Asterisks indicate the level of statistical significance* (^**^*p* < .01, ^*^*p* < .05, ﻿after FDR correction for multiple testing).

Figure S1. Contribution of variables to Dim-1(PC1; 30.86%) and Dim-2 (PC2; 9.86%) in PCA of eating behaviors.

Note: Details on this online questionnaire survey is available from the corresponding authors.
